# *Anopheles* Immune Genes and Amino Acid Sites Evolving Under the Effect of Positive Selection

**DOI:** 10.1371/journal.pone.0008885

**Published:** 2010-01-26

**Authors:** Aristeidis Parmakelis, Marina Moustaka, Nikolaos Poulakakis, Christos Louis, Michel A. Slotman, Jonathon C. Marshall, Parfait H. Awono-Ambene, Christophe Antonio-Nkondjio, Frederic Simard, Adalgisa Caccone, Jeffrey R. Powell

**Affiliations:** 1 Department of Ecology and Taxonomy, Faculty of Biology, National and Kapodistrian University of Athens, Panepistimioupoli Zografou, Athens, Greece; 2 Department of Biology, University of Crete, Heraklion, Crete, Greece; 3 Department of Ecology and Evolutionary Biology, Yale University, New Haven, Connecticut, United States of America; 4 Institute of Molecular Biology and Biotechnology, Foundation of Research and Technology Heraklion, Vassilika Vouton, Heraklion, Crete, Greece; 5 Department of Entomology, Texas A&M University, College Station, Texas, United States of America; 6 Department of Zoology, Weber State University, Ogden, Utah, United States of America; 7 Organisation de Coordination pour la Lutte Contre les Endémies en Afrique Centrale (OCEAC), Yaoundé, Cameroon; 8 Institut de Recherche pour le Développement (IRD), Bobo Dioulasso, Burkina Faso; McGill University, Canada

## Abstract

**Background:**

It has long been the goal of vector biology to generate genetic knowledge that can be used to “manipulate” natural populations of vectors to eliminate or lessen disease burden. While long in coming, progress towards reaching this goal has been made. Aiming to increase our understanding regarding the interactions between *Plasmodium* and the *Anopheles* immune genes, we investigated the patterns of genetic diversity of four anti-*Plasmodium* genes in the *Anopheles gambiae* complex of species.

**Methodology/Principal Findings:**

Within a comparative phylogenetic and population genetics framework, the evolutionary history of four innate immunity genes within the *An. gambiae* complex (including the two most important human malaria vectors, *An. gambiae* and *An. arabiensis*) is reconstructed. The effect of natural selection in shaping the genes' diversity is examined.

Introgression and retention of ancestral polymorphisms are relatively rare at all loci. Despite the potential confounding effects of these processes, we could identify sites that exhibited dN/dS ratios greater than 1.

**Conclusions/Significance:**

In two of the studied genes, *CLIPB14* and *FBN8*, several sites indicated evolution under positive selection, with *CLIPB14* exhibiting the most consistent evidence. Considering only the sites that were consistently identified by all methods, two sites in *CLIPB14* are adaptively driven. However, the analysis inferring the lineage -specific evolution of each gene was not in favor of any of the *Anopheles* lineages evolving under the constraints imposed by positive selection. Nevertheless, the loci and the specific amino acids that were identified as evolving under strong evolutionary pressure merit further investigation for their involvement in the *Anopheles* defense against microbes in general.

## Introduction

It has long been a goal of vector biology to generate genetic knowledge that can be used to “manipulate” natural populations of vectors to ammeliorate the impact of diseases spread by vectors. While long in coming, progress towards reaching this goal has accelerated. Some of the explored methods for generating refractoriness involve using antibodies that kill parasites within the mosquito [1] and discovering genes that govern refractoriness in natural populations [2]. To this end, a great deal is being discovered about the immune system of mosquitoes [3], [4], [5], leading to the hope that the development of an effective gene construct that reduces the ability of mosquitoes to transmit malaria is not far away.

It is clear that species-specific (on both the mosquito and parasite side) interactions guide the co-evolution of *Anopheles* and *Plasmodium*. That *An. gambiae* has undergone an adaptive response to *P. falciparum* infection is suggested by several lines of evidence. Both *An. gambiae* and *An. stephensi* mosquitoes infected with *P. berghei*, for which this parasite species are not natural hosts, produce 50–80 oocysts, whereas an infection with *P. falciparum* results in far fewer oocysts. Furthermore, a specific strain of *An*. *gambiae* selected to be refractory to *P*. *cynomolgi* (monkey malaria) has very limited refractoriness to strains of *P*. *falciparum* isolated in Africa [6]. Genetic crosses between refractory and susceptible strains indicate that different genes are involved in the encapsulation response to different species of *Plasmodium* [7], [8], [9], [10]. A strain of *An*. *stephensi* selected for refractoriness to *P*. *falciparum* transmission showed no detectable resistance to other *Plasmodium* species [11], [12]. Furthermore, the immune response of *An*. *gambiae*, as detected by changes in gene regulation of immune-related genes, is different in response to *P*. *falciparum* and *P*. *berghei* infections [13]. Finally, a gene silencing assay of three immunity genes of *An. gambiae* infected with *P. falciparum*, indicated that the immune response is quite different from that manifested after infection with *P. berghei* [14]. The results of the latter study highlight one more issue, namely the importance of following up discoveries in laboratory model systems with studies on natural parasite–mosquito interactions.

Presently, few studies [15], [16], [17], [18], [19], [20], [21], [22] have investigated the patterns of genetic diversity and the evolution of the *Anopheles* innate immunity genes involved in *Plasmodium* infection. A total number of approximately 65 innate immunity genes have been studied, representing several immunity gene families. Purifying selection was found to be the most common form of selection operating on immune genes [15], [16], [17], [18], [19], [20], [21], [22], whereas a single case of positive selection acting on the lineage leading to *Anopheles arabiensis* was found in *LRIM1* [22]. Investigating selection patterns in a species complex of closely related species such as the *An. gambiae* complex imposes some limitations [18]. These limitations stem mainly from the phylogenetic and population genetic history of the complex [18], [19]. Researchers are concerned with phylogenetic analysis within the complex, and argue that the use of an appropriate outgroup when investigating patterns of selection in *Anopheles* immunity genes is of critical importance as is the level of within species recombination [18], [19]. In an effort to overcome these issues, researchers have applied modifications of well established positive selection methods to *Anopheles* immunity genes that at least partially circumvent these problems [18]. The recently approved genome sequences [23] from 13 more species of *Anopheles* mosquitoes should resolve the outgroup issue. However, until the new *Anopheles* sequences become available, phylogenetic analysis within the *An. gambiae* complex will have to use *An. melas* and *An. merus* sequences as outgroups. The divergence of these species from the *An. gambiae*/*An. arabiensis* clade should be sufficient in the case of genes evolving under strong positive selection [18].

In the present work we investigated patterns of genetic diversity of four anti- *Plasmodium* genes in the *Anopheles gambiae* complex of species, using a population genetics and phylogenetic framework. The complex is composed of seven species, *An. gambiae*, *An. arabiensis*, *An. melas*, *An. merus*, *An. bwambae* and *An. quadriannulatus* A and B. *An. gambiae* and *An. arabiensis* are the two primary African human malaria vectors, whereas *An. melas*, *An. merus* and *An. bwambae* occasionally transmit human malaria locally but do not have sufficiently wide distributions to be considered primary malaria vectors. The species *An. quadriannulatus* (A and B) are zoophilic and are never or rarely exposed to the human malaria parasite *P. falciparum*.

## Results

Four immunity genes were included in the study (Table 1). These genes are *MDL1*, *MDL2*, *CLIPB14* and *FBN8* and all of them have been implicated to be involved in the defense of *Anopheles* against malaria. *MDL1* is composed of 4 exons and has a transcript length of 750 bp. We amplified a fragment ranging from 618 to 739 bp from all specimens (76 sequences, Table 2). This fragment includes the complete sequence of exons one, two and three, part of exon four, and codes for 151 amino acids (Table S1). *MDL2* comprises four exons and has a total length of 3915 bp. The transcript however is only 710 bp long and only 498 bp are translated into amino acids. A total of 49 sequences were obtained from the studied species (Table 2). Sequence length varied from 540 to 868 bp, and included all 498 bp that translate into amino acids (Table S2). *CLIPB14* is composed of three exons. We obtained 49 sequences. The fragments amplified in all species was 1364 bp long, with the exception of the fragments amplified from *An. bwambae*, which were 1271 bp in length. We obtained almost the complete sequence of exon one (except 186 bp in the 5′ end of the exon), and the complete sequence of exon three (Table S3). However, we have not been able to amplify a large fragment (208 bp in *An. bwambae* and 116 in the remaining five species) of the second exon. The sequences amplified translate into a total of 318 amino acids. For the *FBN8* locus we successfully determined 50 sequences from the six species. From this gene, which is not interrupted by an intron (Table 1), we amplified fragments ranging from 512 to 582 bp long. The amplified fragments translate into 194 amino acids (Table S4) out of the 214 residues that constitute the FBN8 protein.

**Table 1:** Details of the loci analyzed and sequences of primers used in the study for the amplification of the immunity genes.

Locus (*Anopheles* chromosome)	Locus length (number of exons)	Length of transcript (bp)	Translation length (aa)	Sequences of primers used	
				Initial PCR	Nested PCR
*MDL1* (3L)	992 bp (4)	750	157	*Intron_248F*: TCTGTTGGCTGCCATGTCAG	*Exon_294F*: CATCACTGTTGGCGTGAGTC
				*Intron_1086R*: TACACGGTCGTCCCACCAGC	*Exon_1013R*: TGGTGATGTTGATCTGCACG
*MDL2* (2R)	3915 bp (4)	710	166	*Intron_073F*: CGCAGATTTTATCCCACGAT	*Exon_092F*: TTCGAGTGGCTACCGAGAGT
				*Intron_944R*: TCATAACCCAACAGCTCACG	*Exon_880R* GACCAGCAGCATGCTATTCA
*CLIPB14* (3L)	1554 bp (3)	1225	389	*Exon1_011F (primer pair A)*: CGGATCGTTTACCACACTGTG	*Exon1_011F (primer pair A)*: CGGATCGTTTACCACACTGTG
				*Exon2_1164R (primer pair A)*: CTGATTCGGTCGAACCCCAG	*Exon2_946R (primer pair A)*: ATTGTACTCCACGTCCGCTG
				*Exon2_1073F (primer pair B)*: GACCGAATCAGGGAAGGAGT	*Intron3_1157F (primer pair B)*: GATTCCCTCCTCCCGAATAG
				*Intron4_1664R (primer pair B)*: TCCTGGCATTTGTATCACCA	*Intron4_1556R (primer pair B)*: TAAACAACTTCCGACGCTCA
*FBN8* (3L)	645 bp (1)	645	214	*Exon_407F*: TCGACGAAAACCCCCGTTCG	-
				*Exon_959R*: CCAACATATAGCTTTTGGGTCCAC	

If the initial PCR was not successful or produced very low signal a nested PCR protocol was implemented.

**Table 2:** Loci polymorphism (coding and non-coding sequence).

	*Locus*																							
	*MDL1*						*MDL2*						*CLIPB14*						*FBN8*					
	*Parameters*																							
	N	n	A	Hd	PiSyn	PiNonS	N	n	A	Hd	PiSyn	PiNonS	N	n	A	Hd	PiSyn	PiNonS	N	n	A	Hd	PiSyn	PiNonS
***Species***																								
*ARA*	8	15	10	0.89	0.011	0.002	6	9	7	0.92	0.014	0.015	6	11	11	1.00	0.019	0.006	5	10	8	0.95	0.064	0.015
*BWA*	6	12	8	0.94	0.008	0.002	7	7	2	0.29	0.012	0.000	5	6	6	1.00	0.020	0.004	4	8	4	0.78	0.021	0.007
*GAM*	7	12	12	1.00	0.027	0.002	6	12	12	1.00	0.023	0.002	6	8	8	1.00	0.013	0.001	5	10	10	1.00	0.068	0.014
*MEL*	7	10	9	0.98	0.008	0.004	6	6	2	0.60	0.000	0.000	6	7	6	0.95	0.005	0.002	5	7	6	0.95	0.062	0.018
*MER*	7	12	12	1.00	0.032	0.004	5	5	5	1.00	0.006	0.001	6	9	9	1.00	0.019	0.004	6	7	3	0.52	0.052	0.016
*QUA/KNP905/SQUA*	9	15	15	1.00	0.025	0.005	10	10	6	0.78	0.010	0.000	6	8	8	1.00	0.012	0.003	5	8	6	0.93	0.081	0.019
*Total n*		*76*						*49*						*49*						*50*				

N: number of individuals, n: number of produced sequences; A: number of unique alleles; Hd: haplotype (allelic) diversity; PiSyn: within species diversity (coding sequence) in synonymous sites; PiNonS: within species diversity (coding sequence) in non-synonymous sites. Species names are abbreviated as follows: *An. arabiensis*: *ARA, An.bwambae*: *BWA*, *An. gambiae: GAM*, *An. melas*: *MEL*, *An. merus*: *MER*, *An. quadriannulatus*: *QUA* (KNP905 and SQUA are also abbreviated as QUA for presentation purposes).

### Diversity, Polymorphism and Phylogeny Inference

**Figure 1:**
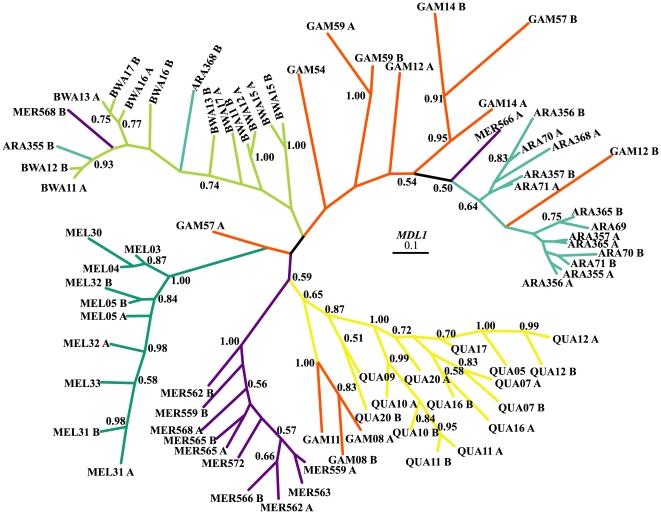
50% majority-rule consensus Bayesian (unrooted) tree of *MDL1*. Numbers on branches are the posterior probabilities of clades, only values above 0.5 are presented. Species names have been abbreviated as follows: ARA: *An. arabiensis*, BWA: *An. bwambae*, GAM: *An. gambiae*, MEL: *An. melas*, MER: *An. merus*, and KNP905: *An. quadriannulatus*. The number following the species abbreviation refers to the individual specimen code, whereas the letters A and B differentiate between the two alleles of a single individual specimen.

Out of the 76 sequences of *MDL1*, 66 different alleles were found (Table 2), and one allele was shared between species (*An. bwambae*-*An. quadriannulatus*). The within species nucleotide diversity (Pi) varied from 0.008 to 0.032 and 0.002 to 0.005 in the synonymous and non-synonymous sites, respectively (Table 2). Dxy (average number of nucleotide substitutions per site between species) ranged from 1.10 to 2.83% (Table S5). The phylogenetic tree of *MDL1* was not fully resolved and multiple polytomies were present (Fig. 1). Nevertheless, *MDL1* was subjected to the maximum likelihood tests for positive selection with PAML and HyPhy.

**Figure 2:**
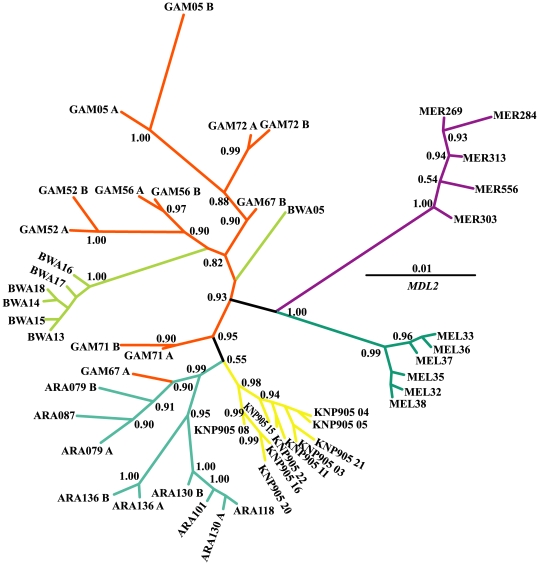
50% majority-rule consensus Bayesian (unrooted) tree of *MDL2*. Numbers on branches are the posterior probabilities of clades, only values above 0.5 are presented. Species names have been abbreviated as follows: ARA: *An. arabiensis*, BWA: *An. bwambae*, GAM: *An. gambiae*, MEL: *An. melas*, MER: *An. merus*, and KNP905: *An. quadriannulatus*. The number following the species abbreviation refers to the individual specimen code, whereas the letters A and B differentiate between the two alleles of a single individual specimen.

Out of the 49 *MDL2* sequences 34 different alleles were detectd (Table 2). No alleles were shared between species. Dxy ranged from 1.10 to 3.02% (Table S5). Within species nucleotide diversity (Pi) ranged between 0.000 to 0.014 and 0.000 to 0.015 in the synonymous and non-synonymous sites, respectively (Table 2). With the exception of one allele from *An. bwambae* and three alleles from *An. gambiae*, all *MDL2* alleles were grouped according to species of origin (Fig. 2).

**Figure 3:**
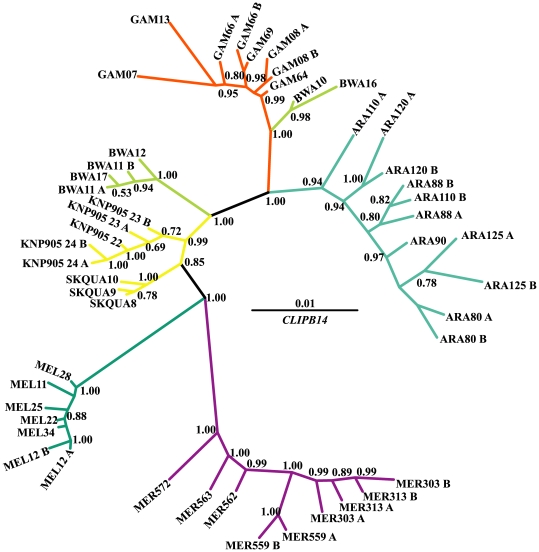
50% majority-rule consensus Bayesian (unrooted) tree of *CLIPB14*. Numbers on branches are the posterior probabilities of clades, only values above 0.5 are presented. Species names have been abbreviated as follows: ARA: *An. arabiensis*, BWA: *An. bwambae*, GAM: *An. gambiae*, MEL: *An. melas*, MER: *An. merus*, and KNP905/SQUA: *An. quadriannulatus*. The number following the species abbreviation refers to the individual specimen code, whereas the letters A and B differentiate between the two alleles of a single individual specimen.

Out of the 49 sequences of *CLIPB14*, 48 alleles were found (Table 2); none were shared between species. Dxy ranged from 1.39 to 3.39% between species (Table S6). The within species nucleotide diversity (Pi) varied from 0.005 to 0.020 in the synonymous sites, and from 0.001 to 0.006 in the non-synonymous sites (Table 2). The alleles of each *Anopheles* species formed a strongly supported monophyletic clade (Fig. 3), with the exception of *An. bwambae*, in which two alleles were closely related to the alleles of *An. gambiae*.

**Figure 4:**
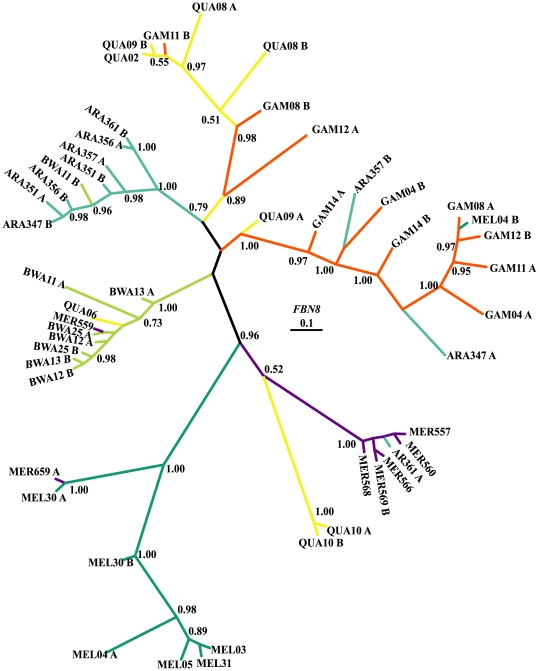
50% majority-rule consensus Bayesian (unrooted) tree of *FBN8*. Numbers on branches are the posterior probabilities of clades, only values above 0.5 are presented. Species names have been abbreviated as follows: ARA: *An. arabiensis*, BWA: *An. bwambae*, GAM: *An. gambiae*, MEL: *An. melas*, MER: *An. merus*, and QUA: *An. quadriannulatus*. The number following the species abbreviation refers to the individual specimen code, whereas the letters A and B differentiate between the two alleles of a single individual specimen.

In *FBN8*, 37 out of the 50 sequences were different alleles (Table 2). Five alleles were shared between species. More specifically, alleles (one in each case) were shared between *An. gambiae* and *An. quadriannulatus*, *An. gambiae* and *An. melas*, *An. arabiensis* and *An. merus*, *An. bwambae* and *An. merus* and *An. melas* and *An. merus*. Dxy ranged from 2.82 to 5.59% between species. Nucleotide diversity within species (Pi) in the synonymous sites was higher compared to the previous genes and varied from 0.021 to 0.081, whereas in the non-synonymous sites ranged from 0.007 up to 0.019. In the *FBN8* tree (Fig. 4) many alleles appear to be more closely related to alleles of species other than their nominal ones. This is particularly the case in *An. quadriannulatus* in which 3 out of the 8 alleles are scattered across the phylogenetic tree and cluster with alleles of *An. merus* and *An. bwambae*. The situation is similar for the *An. gambiae* alleles that appear closely related to *An. quadriannulatus* alleles. Finally, some of the *An. arabiensis* alleles group with *An. gambiae* and *An. merus* alleles.

The *F_ST_* values (permutation tests were significant in all cases) for all studied loci in the majority of the species pairwise comparisons were well above 0.4. In several cases the *F_ST_* exceeded 0.7. However, there were two cases, one in *FBN8* (*An. gambiae*-*An. quadriannulatus*) and one in *MDL1* (*An. gambiae*-*An. quadriannulatus*) where the *F_ST_* values were lower than 0.25.

### Recombination Detection

The analysis using the RDP software did not detect any statistically significant recombination events in the *MDL1* and *MDL2* datasets. In total, four recombination events were detected in the *CLIPB14* and *FBN8* datasets (two events in each locus). The results from the implementation of the GARD module in detecting recombination in the datasets are presented in conjunction with the selection analysis results.

### Results of Selection Analyses Using PAML and HyPhy

In *MDL1* and *MDL2* no sites evolving under positive selection could be inferred, only indications of varying selection pressure (M0 vs. M3) among codons were found (Table 3). The GARD module of HyPhy detected no recombination breakpoints in *MDL1* and *MDL2*, and no sites under positive selection were found by the consecutive analyses of HyPhy as well.

**Table 3:** Likelihood ratio tests (PAML) in *Anopheles* immunity genes between models that allow positive selection (M3, M2a, M8) and those that do not (M0, M1a, M7) and identification of sites exhibiting dN/dS ratio >1.

	Parameter	M0	M3^b^	M1a	M2a^c^	M7	M8^b^
*MDL1*	*-Ln*	-1416.483813	-1408.434119	-1409.626836	-1408.434119	-1411.276475	-1408.462705
	*2ΔLn* a	16.099388		2.385433		5.627539	
	*p-value*	0.003*		0.303		0.060	
	*df*	4		2		2	
	d *Sites exhibiting dN/dS ratio>1*	n.a.	72 (A)^e^, 139(S)	n.a.	none	n.a.	none
*MDL2*	*-Ln*	-989.379352	-978.353695	-978.965428	-978.367537	-979.437982	-978.355848
	*2ΔLn* a	22.051314		1.195782		2.164268	
	*p-value*	0.000*		0.550		0.339	
	*df*	4		2		2	
	d *Sites exhibiting dN/dS ratio>1*	n.a.	6 (T)^e^	n.a.	none	n.a.	none
*CLIPB14*	*-Ln*	-2401.296114	-2336.814182	-2353.547453	-2338.457621	-2354.454745	-2339.391860
	*2ΔLn* a	128.963864		30.179664		30.125770	
	*p-value*	0.000*		0.000*		0.000*	
	*df*	4		2		2	
	d *Sites exhibiting dN/dS ratio>1*	n.a.	4 (C), 10 (K), 46 (V), 52 (Q), 60 (G), **78** (A), 133 (A), **202** (S), 309 (E), 331 (D), 340 (V), 347 (M), 348 (E), 357 (A)	n.a.	**78** (A), **202** (S), 347 (M)	n.a.	4 (C), 60 (G), **78** (A), **202** (S), 347 (M)
*FBN8*	*-Ln*	-1958.216880	-1919.582306	-1924.155342	-1919.618536	-1925.833217	-1919.649726
	*2ΔLn* a	77.269148		9.073612		12.366982	
	*p-value*	0.000*		0.010*		0.002*	
	*df*	4		2		2	
	d *Sites exhibiting dN/dS ratio>1*	n.a.	34 (L), **64** (V), 72 (T), 82 (D), 93 (Y), 107 (V), 130 (T), 140 (H), 165 (Y)	n.a.	34 (L)	-	34 (L), 72 (T)

* Significant *p-value* at 0.05 significance level; *df*: degrees of freedom.

a This quantity is compared to the critical values of a chi-square distribution with the respective degrees of freedom.

b Probability>0.95 in the Naive Empirical Bayes (NEB) analyses of PAML. Even though positive selection based on indications of the NEB analysis is questionable, we are reporting the results for within-gene comparative purposes.

c Probability>0.99 in the Bayes Empirical Bayes (BEB) analyses of PAML.

d Sites refer to amino acid positions of the proteins.

e Letters in parentheses refer to the amino acid present at the site.

Sites in bold characters indicate those that were identified by HyPhy as well.

In *CLIPB14* multiple sites were indicated as having a dN/dS ratio greater than 1; they were identified both by the naive empirical Bayes (NEB) and the Bayes empirical Bayes (BEB) analyses (see Table 3) of PAML. All the sites suggested by the BEB and the NEB analysis as having a dN/dS ratio greater than 1 (probability >0.99) are presented in Table 3. Three of them (78, 202 and 347: Table S3) were consistently identified by all pair wise tests. The dN/dS ratios in these sites were estimated to be well above 1 in all cases, even when considering the standard error of the estimates. Signs of recombination were detected in *CLIPB14* with the GARD module of HyPhy. A single breakpoint was found at position 567 by GARD. Following that, the dataset was partitioned accordingly to take the specific breakpoint into account. At least one of the selection models of the HyPhy analyses, identified sites 78 and 202 as being under positive selection. The estimated dN/dS ratios for these sites by far exceeded those detected by the PAML analysis.

In the *FBN8* gene the maximum likelihood tests of PAML indicated several sites as exhibiting values of dN/dS greater than 1. Two sites were repeatedly identified by the likelihood ratio tests. These sites were 34 and 72 (Table 3, Table S4) and their dN/dS values were greater than 1 in all cases (standard error considered). A single recombination breakpoint was detected by the GARD module of HyPhy at position 277. After partitioning the dataset to avoid the results being affected by recombination, a single site (64) was found to be exhibiting a dN/dS (normalized) value of 3.43.

### Lineage Specific Positive Selection

The branch-site 2 test (PAML) was applied for all the genes with indications of positive selection by any of the preceding tests. In both schemes applied in the branch-site 2 test, and regardless of which species was designated as the foreground branch, the likelihood ratio tests (PAML) were not in favor of positive selection acting on any of the *Anopheles* lineages in any of the genes (results not shown).

### PAML Analyses and Recombination

The RDP analysis detected two sequences as the result of recombination in each one of the *CLIPB14* and *FBN8* datasets. Following that, the recombinant sequences were removed from the respective datasets and a new Bayesian tree was inferred for each locus. These trees were used as input trees in a second series of analyses with PAML. The results of these analyses were identical to the initial one, thus neither the phylogenetic trees nor the PAML outputs are presented separately.

### Selection Analyses Using the McDonald-Kreitman Test

Regarding the McDonald-Kreitman tests, no positive selection acting on any of the studied loci (Tables S5, S6) was detected.

## Discussion

As anticipated the within species diversity is higher in the synonymous sites in all loci. The levels of within species diversity both in the synonymous (PiSyn) and the non-synonymous sites (PiNonSyn) are significantly higher in the *FBN8* locus in all six species (Fig. S1, S2). The other loci exhibit similar levels of within species diversity in the synonymous and the non-synonymous sites. An exception is the level of non-synonymous diversity within *An. arabiensis* in *MDL2*, which matches that of *FBN8* (Table 2, Fig. S1, S2). The levels of divergence of *MDL1* and *MDL2* between the different species (Dxy) are in the same range, having a mean value of 1.96 and 2.00%, respectively (Table S5). The between species divergence is higher in *CLIPB14* compared to *MDL1* and *MDL2* with a mean value of 2.56%, whereas this value is 4.20% for *FBN8* (Table S6). The levels of divergence recorded both within and between the species for the four studied loci, are comparable to those that have been estimated for other innate immunity genes of *Anopheles* [19], [20], [22].

In all four loci some alleles were shared between species. This is an indication that introgression and/or retention of ancestral polymorphisms have affected the distribution of the genetic diversity of these immune genes within the *Anopheles gambiae* complex. This is also evident from the fact that in all studied loci, some alleles are more closely related to alleles other than their nominal ones. To evaluate the effect of introgression and/or retention of ancestral polymorphisms, we have estimated *F_ST_* values between the species of the complex for each locus separately. The *F_ST_* values reported herein are within the range reported for other *Anopheles* innate immunity genes [19] and indicative of great genetic differentiation between the species of the complex. It is very important to note at this point that allele sharing between species is restricted to a single allele in *MDL1*, and 5 alleles in *FBN8* out of a total of 185 alleles detected at all four loci. Furthermore, no shared alleles were detected between *An. gambiae s.s.* and *An. arabiensis*. It seems that allele sharing and retention of ancestral polymorphisms is present only at a very low level, at least for the four genes we studied. Therefore, all the species are significantly differentiated (Tables S5, S6) with no or very little indication of gene flow for these four loci (F_ST_ values and Tables S5, S6). This degree of differentiation and isolation minimizes the confounding effects of these processes in the investigation of selection patterns.

In all the studied loci relatively few fixed non-synonymous differences (Tables S5, S6) were found and the McDonald-Kreitman tests did not detect positive selection acting on any of them. We concur with [18] that this is probably due to the limitations relating to our datasets in conjunction with the inherent properties of the McDonald-Kreitman test.

Regarding *MDL1*, the result of the McDonald-Kreitman test is corroborated by the maximum likelihood tests implemented in the PAML and HyPhy analysis.

Similarly to *MDL1*, *MDL2* was also found to be evolving under purifying selection. This gene has been shown to exhibit significant induction in the midgut tissue upon *P. falciparum* ookinete invasion. However, it did not show specificity to *P. falciparum* infection [3]. At the same time, the maximum likelihood tests of PAML and HyPhy did not detect any branches and/or sites evolving under positive selection.

In *CLIPB14*, both the PAML and the HyPhy analyses suggest that there may be specific sites of the CLIPB14 protein that exhibit a dN/dS ratio greater than 1. Sites 78 and 202, were identified as exhibiting a dN/dS ratio above 1 in both analyses.

In *FBN8* sites 34 and 72 were identified by the BEB method as possibly being under positive selection. However, these sites were not verified by the HyPhy analysis. Site 64, which was identified by the NEB analysis of PAML based on the M3 model (Table 3), was also identified by HyPhy with a dN/dS value of 3.43.

The level of recombination detected in our datasets is below the threshold level suggested by Anisimova *et al*., (2003), above which recombination may be mistaken for molecular adaptation. Despite this, the PAML analyses for *CLIPB14* and *FBN8*, in which signs of positive selection were indicated, were re-run after the exclusion of the recombinant sequences. The second series of the PAML analyses, after the exclusion of the recombinant sequences, indicated by the RDP software, verified the results of the initial analyses in both genes. We consider this an analysis scheme that significantly reduces the effect of recombination in the inference of selection patterns acting on the datasets.

From the points just made, we conclude that the data collected and methods of analysis implemented, were adequate to detect selection for those genes undergoing positive selection. The analyses we used were capable of overcoming problems posed by evolutionary processes such as introgression, ancestral polymorphisms or recombination. It is not surprising, and it is indeed encouraging, that only a minority of the innate immunity genes that have been studied in this way [16], [20], [22] exhibit signs of positive selection. The “arsenal” of immunity genes is more than 100 [24] known genes and the fact that the approach we used can eliminate several, and yet detect positive signals for a minority of them is to be expected and gives us hope that these are not false positives. That is, the approach followed here has the power to identify negatives and positives between a set of immunity genes that have multiple roles in the defense of *Anopheles* against pathogens. Very strong indications exist for their involvement in the immune response of *Anopheles* against *Plasmodium*, whereas at the same time they are found to be part of an immunity cascade towards bacteria.

Even though specific codons (two in *CLIPB14* and two in *FBN8*), were found to be exhibiting dN/dS ratios greater than one using PAML, we did not detect positive selection acting on specific lineages involving malaria vectors. This may simply reflect the limitation of the data and the sensitivity of the analytical procedures implemented. Nevertheless,variation at these sites is consistent with an influence on *Plasmodium* infection.

In conclusion, considering only the sites that were consistently identified by all methods applied, two in the *CLIPB14* locus have the most evidence of being adaptively driven. These are sites 78 and 202. Understanding the biological mechanisms underlying this positive selection is beyond the scope of this work. On the other hand, in *FBN8* sites 34 and 72 that were identified by the PAML analyses were not verified by the HyPhy results. Therefore, caution should be exerted regarding this locus and the specific amino acid sites. However, we do believe that these sites could also serve as a starting point for geneticists wishing to genetically manipulate *Anopheles* immunity genes. In most published sequences [25] positively selected sites inferred by the BEB method of PAML were found to be biologically meaningful following a 3D structure study of the proteins. Perhaps a similar approach could be applied to the CLIPB14 and FBN8 proteins and reveal the role of the positively selected sites in the proteins' structures implying function and their possible involvement in the defense against *Plasmodium* and/or other microbes.

## Materials and Methods

### Mosquitoes Samples

Six species of the *An. gambiae* complex were used: *An. gambiae* sensu stricto, *An. arabiensis*, *An. bwambae*, *An. melas*, *An. merus* and *An. quadriannulatus*. Details on the origin of specimens and DNA extraction methods are provided by [20]. Species names in figures and tables are abbreviated as follows: *An. arabiensis*: ARA, *An.bwambae*: BWA, *An. gambiae*: GAM, *An. melas*: MEL, *An. merus*: MER, *An. quadriannulatus*: QUA/KPN905/SQUA (depending on location of origin).

### Analysis of Immunity Genes

A detailed description of the analyzed loci is given in Table 1. Among the analyzed genes are *MDL1* (Ensembl Gene Id: AGAP012352) and *MDL2* (Ensembl Gene Id: AGAP002857). Both genes encode an MD-2-like protein and belong to a 13-member gene family [3]. The expression of both was induced in midgut tissue upon *P. falciparum* ookinete invasion. Furthermore, in RNAi gene silencing assays *MDL1* showed specificity in regulating mosquito resistance in *P. falciparum* but not in *P. berghei*.

*CLIPB14*, a gene encoding a clip domain serine protease (Ensemble Gene Id: AGAP010833) was also included in the study. This gene is expressed in mosquito hemocytes and is transcriptionally induced by both bacterial and *Plasmodium* challenges [26]. Functional studies applying RNA interference revealed that *CLIPB14* is involved in the elimination of *Plasmodium* ookinetes in *An. gambiae* [26]. Finally, we included a member of the fibrinogen-related protein (FREP) genes, *FBN8* (Ensemble Gene Id: AGAP011223). The fibrinogen-like (FBG) domains in members of this protein family, are predicted to recognize carbohydrates and their derivatives on the surface of microorganisms during the innate immune response [27]. As pointed out by [27], the ability of mosquitoes to recognize parasites in innate immunity and physiologies associated with blood feeding, is probably correlated with the structure of the FBG domains.

Besides the above mentioned reasons for selecting the specific set of immunity genes, we have to note that these loci were also investigated by [3] as well, and scored relatively high regarding their specific involvement in the defense against *Plasmodium*.

Multiple primers were designed for each of the targeted loci based on the *An. gambiae* genome [28]. Primers were manually designed and their characteristics were estimated using FastPCR [29]. This software was also used to investigate potential primer-pair incompatibilities. A nested-PCR protocol was used to amplify *MDL1*, *MDL2* and *CLIPB14*. The sequences of the primers used in the amplification of each locus are shown in Table 1. PCR products were separated electrophoretically on a 1–2% agarose gel, purified using commercially available kits, and were sequenced in both directions in a 3730 ABI capillary sequencer. All individuals that were found to be heterozygous for two or more positions were re-amplified purified and cloned using the TOPO-TA cloning kit for sequencing (Invitrogen). From each individual, a minimum of three transformed colonies were selected, and the size of the DNA insert was determined by PCR using the T3/T7 primer pair of the TOPO-TA vector. In cases where the size of PCR product indicated the presence of the correct insert, this product was sequenced in both directions. To ensure a minimal number of miss-incorporations, Platinum High Fidelity Taq (Invitrogen) was used in all amplifications. Sequence chromatograms were inspected by eye to confirm differences between alleles of the same individual, or within and between species. Sequences were viewed, edited, assembled and aligned using CodonCode Aligner (v.1.6.3 CodonCode Corporation, Dedham, MA, USA). All sequences were blasted using the BLAST tool of VectorBase (http://www.vectorbase.org/Tools/BLAST) against the *An. gambiae* genome to verify homology to the respective loci. The sequences have been submitted to GenBank under the accession numbers GU432776 to GU432999.

### Polymorphism and Divergence

Basic analyses of polymorphism and divergence were performed using the computer program DNAsp [30]. Estimated parameters included the within-species pairwise diversity (Pi) at synonymous and non-synonymous sites, and the average number of nucleotide substitutions per site between species (Dxy). Introgression and/or retention of ancestral polymorphisms have resulted in the sharing of variation between the *An. gambiae* complex members [22], [31], [32], [33] and the calculation of *F_ST_* between the members of the complex therefore becomes meaningful [31]. We used DNAsp [30] to calculate *F_ST_* values between the six species. *F_ST_* values measure the genetic differentiation as a proportion of total diversity that is due to between-group differences.The permutation test (5000 replicates) as implemented in DNAsp, was applied to address the question of whether the observed *F_ST_* values are significantly greater than zero.

### Detecting Genes Affected by Positive Selection Using PAML

Phylogenies were constructed using both coding and non-coding regions for each of the four genes with MrBayes 3.1 [34], using partitioned or non-partitioned data depending on the dataset. In partitioned datasets a different substitution model was applied to the introns, the first, second, and third codon positions. The substitution models were suggested by Modeltest 3.7 [35] according to the Akaike Information Criterion [36].

These Bayesian trees were used to implement the maximum likelihood methods of the PAML v.4. package [37] aimed at detecting codons that show signs of adaptive evolution. For each locus, datasets were initially analyzed using the M0 (one-ratio) model implemented in the “codeml” program. The M0 model assumes a constant ω value (dN/dS ratio) along all branches in the tree and among all codon sites in the gene [37]. At least two runs of the M0 model were performed on each alignment to check the consistency of the log-likelihood values between the multiple runs. Runs that were not consistent were rerun until the values converged. In the subsequent calculations of the log-likelihood of each tree under the M1a, M2a, M7 and M8 models of PAML, the initial branch lengths were those estimated under the M0 model. Model M1a is a neutral model which divides the codon sites into two categories, one having conserved sites with ω_0_ = 0 and the other involving neutral sites with ω_1_ = 1. Model M2a allows an additional category of sites with ω_2_ estimated from the data, thus accommodating positively selected sites. Models M7 and M8 assume that ω follows a beta distribution with the shape parameters estimated in the interval (0, 1), and M8 includes one additional category to account for positively selected sites [38]. In all these models the rate of synonymous substitutions (dS) is constant among sites, while the rate of non-synonymous substitutions (dN) is variable [39]. According to [37], the site model pairs that appear to be particularly useful for real data analysis, are M1a versus M2a, and M7 versus M8. The significance of positive selection was calculated by comparing twice the log likelihood difference in a chi-square test with two degrees of freedom.

The branch-site 2 test [40], [41] as implemented in PAML was used to test for positive selection along specific branches. We tested each of the *Anopheles* clades on the species phylogeny, treating each in turn as the foreground clade. The alternative branch-site model has four codon site categories; two for sites evolving under purifying and neutral selection along branches, and two for sites under positive selection along the foreground branch. The null model restricts sites on the foreground lineage to evolve neutrally. Each branch-site model was run multiple times to ensure convergence of log-likelihood values. The significance of positive selection was calculated by comparing twice the log-likelihood difference in a chi-square test with 1 degree of freedom. Since some species appeared as non-monophyletic in the phylogenetic analyses, two different running schemes were implemented for the branch-site 2 test. In the first scheme all alleles, even those of a different species (non-species alleles), that clustered within a specific *Anopheles* species clade were assigned to the foreground branch. In the second scheme, the non-species alleles were not assigned to the foreground branch.

In PAML the NEB method [25], [39] and the BEB method [42] are used to identify sites under positive selection. BEB is implemented under models M2a and M8 only. As suggested in the PAML manual only the results of the BEB method should be considered robust in the identification of sites under selection.

### Implementing HyPhy in the Detection of Positive Selection and GARD to Screen for Recombination

To verify whether the codon sites inferred by PAML to be under positive selection are identified by other methods as well, the program HyPhy [43] was implemented using Datamonkey [44], the Web interface of HyPhy. Similarly to PAML, the likelihood methods in HyPhy are based on a codon substitution model [45]. Three different codon-based maximum likelihood methods, SLAC, FEL and REL, can be used to estimate the dN/dS (ω) ratio at every codon in the alignment. A detailed discussion of each approach can be found in Pond & Frost [46].

In HyPhy all methods can take recombination into account, provided that prior to the selection analysis a screening of the sequences for recombination breakpoints is performed. This is done by using the GARD module [47]. In the case that recombination breakpoints have been detected the dataset is partitioned and each partition is allowed to have its own phylogenetic tree. Following that, the selection analysis is performed separately on each tree. The HyPhy approach allowed us to investigate whether the presence of recombination in the datasets was producing false positives in the PAML analyses. In contrast to codeml models, HypHy also estimates the rate of synonymous substitutions (dS) at each codon site, thus taking into account potential synonymous rate variation among sites [48]. Furthermore, the PARRIS method [49] of HyPhy, that extends traditional codon-based likelihood ratio tests to detect if a proportion of sites in the alignment evolve with dN/dS>1, was also applied to the datasets. The PARRIS method also takes recombination and synonymous rate variation into account.

The starting tree of each locus that served as the basis for the HyPhy analyses was inferred by the program itself.

### McDonald-Kreitman Test and Positive Selection

A different approach to detect signs of selection involved the McDonald-Kreitman test [50]. This test was applied to our datasets using DNAsp [30]. The McDonald-Kreitman test compares the ratios of fixed to polymorphic substitutions of non-synonymous and synonymous substitutions between species. Under neutrality, the fixation rate of synonymous and non-synonymous substitutions is expected to be equal, but positive selection would increase the rate of fixation in non-synonymous sites. In contrast to the phylogeny-based tests mentioned above, the McDonald-Kreitman test allows the detection of selection on a whole protein, is bound to be quite conservative in detecting selection [51] and lacks the power of a site by site analysis.

### Addressing the Recombination Issue

As already mentioned, within-population recombination can have a confounding effect in the inference of adaptive evolution [52], [53]. Patterns of genetic variability created by recombination can closely resemble the effects of molecular adaptation (e.g. [54]). Current codon models of heterogeneous dN/dS ratios among sites assume no recombination, raising concerns about the possibility that the likelihood ratio tests (LRTs) can mistakenly interpret the effects of recombination as evidence for positive selection. However, as [53] have shown using simulated data, when the recombination events are maintained at a low level (fewer than three recombination events in the history of a sample of ten sequences) the positive selection inferring tests, including the LRT, are quite robust. Furthermore, according to the same authors identification of sites under positive selection by the BEB method [42] appears to be less affected than the LRT by recombination.

In this study, two different approaches were applied to account for the effects of recombination. In the first approach the datasets were analyzed (see section above) using the Web interface of HyPhy, namely Datamonkey [44] and applying the GARD algorithm [47]. In the second approach, the datasets were scanned for recombination using the software RDP [55] and implementing all seven tests included in the package. The settings of the scans in RDP were adjusted according to the software's manual (http://darwin.uvigo.es/rdp/rdp.html). Only the statistically significant recombination events detected by any of the tests of RDP, were considered in the consecutive phylogeny-based analyses.

## Supporting Information

Table S1 MDL1 protein alignment
(0.04 MB XLS)

Table S2 MDL2 protein alignment
(0.03 MB XLS)

Table S3 CLIPB14 protein alignment
(0.05 MB XLS)

Table S4 FBN8 protein alignmnent
(0.04 MB XLS)

Table S5 MacDonald-Kreitman tests on MDL1 and MDL2 and between species divergence (Dxy)
(0.05 MB DOC)

Table S6 MacDonald-Kreitman tests on CLIPB14 and FBN8 and between species divergence (Dxy)
(0.05 MB DOC)

Figure S1 Within species diversity (coding sequence) in synonymous sites (PiSyn) in each locus
(0.20 MB EPS)

Figure S2 Within species diversity (coding sequence) in non-synonymous sites (PiNonSyn) in each locus
(0.20 MB EPS)
