# Supplementary material for: Anopheles Immune Genes and Amino Acid Sites Evolving Under the Effect of Positive Selection
Source: PLoS One. 2010 Jan 26;5(1):e8885. doi: 10.1371/journal.pone.0008885 (PMC2811201; doi:10.1371/journal.pone.0008885)
Supplement: Table S5 — MacDonald-Kreitman tests on MDL1 and MDL2 and between species divergence (Dxy) (0.05 MB DOC) [file pone.0008885.s005.doc]

|  | *MDL1* | | | | | | |  | *MDL2* | | | | | | |
| --- | --- | --- | --- | --- | --- | --- | --- | --- | --- | --- | --- | --- | --- | --- | --- |
|  | Fixed | |  | Polymorp. | |  | Dxy (%) |  | Fixed | |  | Polymorp. | |  | Dxy (%) |
|  | S | NS |  | S | NS | p-value |  |  | S | NS |  | S | NS | p-value |  |
| *ARA-BWA* | 0 | 0 |  | 8 | 5 | - | 1.77 |  | 1 | 0 |  | 9 | 0 | - | 2.19 |
| *ARA-GAM* | 0 | 0 |  | 11 | 7 | - | 1.95 |  | 0 | 0 |  | 11 | 4 | - | 1.45 |
| *ARA-MEL* | 3 | 1 |  | 6 | 7 | n.s. | 2.48 |  | 3 | 0 |  | 5 | 1 | - | 2.01 |
| *ARA-MER* | 0 | 0 |  | 14 | 10 | - | 1.35 |  | 2 | 1 |  | 6 | 2 | n.s. | 2.68 |
| *ARA-QUA* | 0 | 0 |  | 9 | 10 | - | 1.58 |  | 1 | 0 |  | 7 | 0 | - | 1.14 |
| *BWA-GAM* | 1 | 0 |  | 13 | 5 | - | 1.94 |  | 0 | 0 |  | 13 | 0 | - | 1.53 |
| *BWA-MEL* | 3 | 1 |  | 6 | 8 | n.s. | 2.13 |  | 3 | 0 |  | 4 | 0 | - | 2.43 |
| *BWA-MER* | 0 | 0 |  | 16 | 8 | - | 1.87 |  | 4 | 0 |  | 6 | 0 | - | 3.02 |
| *BWA-QUA* | 0 | 0 |  | 10 | 9 | - | 1.10 |  | 0 | 0 |  | 6 | 0 | - | 1.66 |
| *GAM-MEL* | 4 | 1 |  | 11 | 9 | n.s. | 2.52 |  | 2 | 0 |  | 9 | 4 | - | 1.84 |
| *GAM-MER* | 0 | 0 |  | 21 | 9 | - | 2.28 |  | 1 | 0 |  | 7 | 3 | - | 2.55 |
| *GAM-QUA* | 0 | 0 |  | 15 | 10 | - | 1.66 |  | 0 | 0 |  | 11 | 2 | - | 1.18 |
| *MEL-MER* | 3 | 2 |  | 16 | 2 | n.s. | 2.83 |  | 3 | 1 |  | 2 | 1 | n.s. | 2.43 |
| *MEL-QUA* | 3 | 1 |  | 10 | 13 | n.s. | 2.09 |  | 2 | 0 |  | 2 | 0 | - | 1.10 |
| *MER-QUA* | 0 | 0 |  | 20 | 13 | - | 1.83 |  | 3 | 0 |  | 5 | 1 | - | 2.41 |
| Mean value |  |  |  |  |  |  | 1.96 |  |  |  |  |  |  |  | 1.97 |

Species names are abbreviated as in Table 2. S: synonymous mutations, NS: non-synonymous mutations, n.s.: non significant.
