# Supplementary material for: Anopheles Immune Genes and Amino Acid Sites Evolving Under the Effect of Positive Selection
Source: PLoS One. 2010 Jan 26;5(1):e8885. doi: 10.1371/journal.pone.0008885 (PMC2811201; doi:10.1371/journal.pone.0008885)
Supplement: Table S6 — MacDonald-Kreitman tests on CLIPB14 and FBN8 and between species divergence (Dxy) (0.05 MB DOC) [file pone.0008885.s006.doc]

|  | *CLIPB14* | | | | | | |  | *FBN8* | | | | | | |
| --- | --- | --- | --- | --- | --- | --- | --- | --- | --- | --- | --- | --- | --- | --- | --- |
|  | Fixed | |  | Polymorp. | |  | Dxy (%) |  | Fixed | |  | Polymorp. | |  | Dxy (%) |
|  | S | NS |  | S | NS | p-value |  |  | S | NS |  | S | NS | p-value |  |
| *ARA-BWA* | 0 | 1 |  | 18 | 12 | n.s. | 1.87 |  | 0 | 0 |  | 24 | 27 | - | 2.82 |
| *ARA-GAM* | 1 | 0 |  | 20 | 9 | n.s. | 1.62 |  | 0 | 0 |  | 35 | 28 | - | 3.87 |
| *ARA-MEL* | 8 | 6 |  | 15 | 12 | n.s. | 3.34 |  | 0 | 0 |  | 33 | 32 | - | 5.58 |
| *ARA-MER* | 1 | 4 |  | 23 | 17 | n.s. | 3.39 |  | 0 | 0 |  | 29 | 29 | - | 4.07 |
| *ARA-QUA* | 2 | 3 |  | 17 | 11 | n.s. | 2.01 |  | 0 | 0 |  | 32 | 30 | - | 3.73 |
| *BWA-GAM* | 0 | 2 |  | 18 | 8 | n.s. | 1.60 |  | 1 | 0 |  | 27 | 22 | - | 3.62 |
| *BWA-MEL* | 9 | 3 |  | 12 | 10 | n.s. | 2.82 |  | 1 | 0 |  | 28 | 25 | n.s. | 5.36 |
| *BWA-MER* | 4 | 5 |  | 19 | 15 | n.s. | 3.09 |  | 0 | 0 |  | 23 | 25 | - | 3.65 |
| *BWA-QUA* | 1 | 0 |  | 16 | 10 | n.s. | 1.39 |  | 0 | 0 |  | 25 | 23 | - | 3.31 |
| *GAM-MEL* | 11 | 8 |  | 14 | 7 | n.s. | 3.38 |  | 0 | 0 |  | 34 | 26 | - | 5.59 |
| *GAM-MER* | 6 | 7 |  | 20 | 14 | n.s. | 3.55 |  | 1 | 0 |  | 34 | 28 | n.s. | 4.61 |
| *GAM-QUA* | 4 | 5 |  | 16 | 7 | n.s. | 2.07 |  | 0 | 0 |  | 31 | 25 | - | 3.51 |
| *MEL-MER* | 7 | 6 |  | 13 | 15 | n.s. | 3.19 |  | 0 | 0 |  | 25 | 23 | - | 4.92 |
| *MEL-QUA* | 6 | 3 |  | 10 | 9 | n.s. | 2.20 |  | 0 | 0 |  | 30 | 29 | - | 5.36 |
| *MER-QUA* | 3 | 5 |  | 17 | 16 | n.s. | 2.53 |  | 0 | 0 |  | 25 | 26 | - | 4.10 |
| Mean value |  |  |  |  |  |  | 2.54 |  |  |  |  |  |  |  | 4.27 |

Species names are abbreviated as in Table 2. S: synonymous mutations, NS: non-synonymous mutations, n.s.: non significant.
